# Prevalence of co-existent neoplasia in clinically diagnosed pterygia in a UK population

**DOI:** 10.1038/s41433-023-02594-w

**Published:** 2023-05-26

**Authors:** Hibba Quhill, Tejal Magan, Caroline Thaung, Mandeep S. Sagoo

**Affiliations:** 1 Ocular Oncology Service, Moorfields Eye Hospital NHS Foundation Trust, London, UK; 2 Ocular Oncology Service, Sheffield Teaching Hospitals NHS Foundation Trust, Sheffield, UK; 3 Department of Eye Pathology, UCL Institute of Ophthalmology, London, UK; 4 NIHR Biomedical Research Centre for Ophthalmology at Moorfields Eye Hospital and UCL Institute of Ophthalmology, London, UK

**Keywords:** Eye cancer, Risk factors, Conjunctival diseases

## Abstract

**Introduction:**

Ocular surface squamous neoplasia (OSSN) and pterygia share risk factors and co-exist in only a minority of cases. Reported rates of OSSN in specimens sent as pterygium for histopathological analysis vary between 0% and nearly 10%, with the highest rates reported in countries with high levels of ultraviolet light exposure. As there is a paucity of data in European populations, the aim of this study was to report the prevalence of co-existent OSSN or other neoplastic disease in clinically suspected pterygium specimens sent to a specialist ophthalmic pathology service in London, United Kingdom.

**Methods:**

We performed a retrospective review of sequential histopathology records of patients with excised tissue submitted as suspected “pterygium” between 1997 and 2021.

**Results:**

In total, 2061 specimens of pterygia were received during the 24-year period, with a prevalence of neoplasia in those specimens of 0.6% (*n* = 12). On detailed review of the medical records of these patients, half (*n* = 6) had the pre-operative clinical suspicion of possible OSSN. Of those cases without clinical suspicion pre-operatively, one was diagnosed with invasive squamous cell carcinoma of the conjunctiva.

**Conclusion:**

In this study, rates of unexpected diagnoses are reassuringly low. These results may challenge accepted dogma, and influence future guidance for the indications for submitting non-suspicious pterygia for histopathological analysis.

## Introduction

The prevalence of pterygium has been reported to be between 0.3–12%, with highest rates in countries with dry climates and increased ultraviolet B exposure (UVB) [1, 2]. Both pterygia and ocular surface squamous cell neoplasia (OSSN) share common risk factors, including UVB exposure, human papilloma virus infection and chronic ocular surface inflammation [3, 4]. Unsurprisingly, a number of studies have found co-existent OSSN within clinically diagnosed pterygia submitted for histopathological examination [5–16]. The rates of co-existent disease appear to vary by geographic location, perhaps reflecting the prevalence of the shared risk factors. For example, a histopathological diagnosis of OSSN in pterygia specimens was reported to be 0% in Canada and Israel, 0.3–1.7% in the United States and as high as 9.8% in Australia [5–9]. Some studies have shown higher rates than expected given the geographic location [16].

To our knowledge, there is a lack in the literature of studies evaluating the rates of co-existent or incidental OSSN in clinically diagnosed pterygia in European populations, specifically the United Kingdom. We investigated the prevalence of OSSN or other neoplasms in surgically excised pterygia submitted for routine histopathological examination to a single specialist ophthalmic pathology service in London, UK, and provide detailed analysis of those cases with invasive disease.

## Methods

This was a retrospective study of electronic histopathology records from excised pterygia sent to the Department of Eye Pathology (a National Specialist Ophthalmic Pathology Service), University College London Institute of Ophthalmology between January 1997 and June 2021. Specimens had been submitted from Moorfields Eye Hospital NHS Foundation Trust, London, and Moorfields Private Eye Hospital, London. Cases were identified by electronic database search using the keyword “pterygium” and its derivatives in the clinical field on the histopathology request form. Reports sent for cancer registration, or containing the keywords “OSSN”, “squamous”, “dysplasia”, “in situ”, “carcinoma”, “conjunctival intraepithelial neoplasia (CIN)”, “CIN1”, “CIN2”, “CIN3”, “invasive”, “atypia”, “melanoma” or their derivatives were further identified and examined. Cases were excluded if an OSSN diagnosis had been made on a previous biopsy from the same eye. Those cases of clinically suspected pterygia with co-existent OSSN or other malignant diagnoses underwent detailed retrospective medical note review. Cases were scrutinized to detect clinical suspicion of a diagnosis other than pterygium pre-operatively, or the presence of atypical clinical features.

## Results

A total of 2061 specimens of lesions identified clinically as “pterygium”, were submitted for histopathological examination during the almost 25-year inclusion period. In total, 99.4% (*n* = 2049) of cases were simple pterygia without co-existent OSSN. Twelve cases (0.6%) had co-existent neoplasia detected, of which 50% (*n* = 6) were listed without clinical suspicion of atypia. Demographic information and details of all 12 cases with co-existent neoplasia are detailed in Table 1. The three cases with co-existent invasive disease are further described.

**Table 1:** Demographic and clinical details of all 12 cases with co-existent neoplasia on histopathological analysis.

Patient number	Age (years)	Sex	Ethnicity	Risk factors and co-morbidities	Laterality	Location of lesion	Clinical atypia pre-operatively	Details of clinical suspicion	Excision with oncology precautions	Histological diagnosis	Adjuvant treatment	Recurrence
**1**	68	M	Caucasian	Rheumatoid arthritis, systemic lymphoma	L	T	Yes	Vascular, brown pigment	Yes	Invasive melanoma	Strontium brachytherapy	No
**2**	54	M	Asian	Type 2 Diabetes Mellitus	L	T	No	N/A	No	Invasive SCC	N/A	Yes
**3**	64	M	Black	N/A	L	N	Yes	Vascular, thickened gelatinous	Yes	Invasive SCC	Topical MMC and strontium brachytherapy	No
**4**	72	M	Black	Foundry worker	L	I	Yes	Corneal involvement	No	CIN 1	Excision	No
**5**	64	M	Hispanic	N/A	R	N	No	N/A	No	CIN 1	N/A	No
**6**	76	M	Asian	Type 2 Diabetes Mellitus	L	N	Yes		No	CIN 2	N/A	
**7**	34	F	Asian	N/A	L		No	N/A	No	CIN 2	N/A	
**8**	50	F	Black	Atopic conjunctivitis	L	N	Yes	Keratinization, corneal involvement	No	CIN 2	N/A	
**9**	65	M	Caucasian	UV exposure, SCC of contralateral eye	L	N	No	N/A	No	CIN 2	N/A	
**10**	71	M		N/A	L		No	N/A	No	CIN 2	N/A	
**11**	48	M	Caucasian	UV exposure	R		No	N/A	No	CIN 3	Topical interferon	
**12**	57	F		UV exposure, skin SCC	L	N	Yes	Corneal involvement	No	CIN 3	Excision, cryotherapy	

Empty fields indicate missing data.

*M* male, *F* female, *R* right, *L* left, *T* temporal, *N* nasal, *I* inferior, *SCC* squamous cell carcinoma, *CIN* conjunctival intraepithelial neoplasia, *N/A* not applicable, *MMC* mitomycin C.

### Patient number 1

**Fig. 1:**
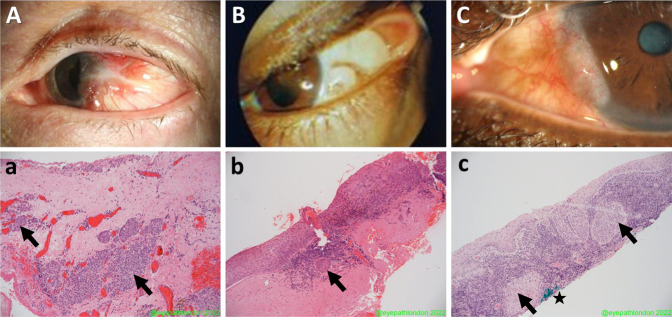
Pre-operative clinical photographs and histopathology of excised specimens of patients with invasive neoplasia. **A** Anterior segment pre-operative photograph of Patient number 1. **a** Histology of excised lesion in (**A**), stained with hematoxylin and eosin (H & E) at low magnification, showing intraepithelial and deeper stromal nests of atypical cells (black arrows) which stain with Melan A and HMB45 (not shown), indicating invasive malignant melanoma. **B** Anterior segment pre-operative photograph of Patient number 2.  **b** Histology of lesion (**B**), stained with H & E at low magnification, showing a traumatized specimen with full thickness disorganization of conjunctival epithelium and a single nest of invasive disease (black arrow) – consistent with early invasive squamous cell carcinoma (SCC). **C** Anterior segment pre-operative photograph of Patient number 3. **c** Histology of the excised lesion in (**C**), stained with H & E at low magnification, showing conjunctival stromal infiltration by inflammatory cells and multiple nests of atypical cells (black arrows), involving the deep margin (inked in green, ★) indicating incompletely excised invasive SCC.

A Caucasian male in his 60s with no relevant past medical history. He had been previously diagnosed with a left temporal pterygium, which had been excised elsewhere 25 years ago. This recurred and was excised again 5 years previously. Histology was reported as benign. He presented with a further recurrence of the lateral bulbar conjunctival lesion which crossed the limbus and involved the cornea (Fig. 1A). The lesion was considered atypical by the treating ophthalmologist due to its location, the presence of fine scattered brown pigment within the lesion and its high vascularity. The lesion was treated as a likely neoplasm with wide excision, cryotherapy to the margins and a “no-touch” technique, taking ocular surface oncological precautions. Histopathology revealed a diagnosis of invasive melanoma (pT1a) arising from a pre-existing compound melanocytic nevus (Fig. 1a). The patient was subsequently treated with adjuvant strontium plaque brachytherapy (fractionated 50 Gy dose) and remains recurrence free to date.

### Patient number 2

A South Asian male in his 50s with no relevant past medical history, presented with a temporal bulbar conjunctival pale nodule with recurrent inflammation but without corneal involvement, on a background of complexion-associated melanosis (Fig. 1B). The treating ophthalmologist considered the lesion to be pterygium or pinguecula and excised the lesion without wide margins or adjuvant therapy. Histopathology revealed early invasive squamous cell carcinoma on a background of severe dysplasia—the invasive portion was completely excised but the dysplastic areas were not (Fig. 1b). The patient did not require adjuvant treatment as the invasive portion was considered to be completely excised. The patient underwent close monitoring, but unfortunately a recurrence was detected three years later. This was treated with wide excision and adjuvant topical mitomycin C. He remains further recurrence free at 14 years follow-up.

### Patient number 3

A Black male in his 60s with no past medical history presented with irritation and redness of the nasal aspect of his left eye. The lesion was vascular and had a thickened “gelatinous” portion crossing the limbus onto the nasal cornea (Fig. 1C). The listing ophthalmologist clinically diagnosed the lesion as a possible pterygium or OSSN, so it was surgically managed as a neoplasm with wide margin surgical excision and cryotherapy (oncology precautions). Histopathology revealed early invasive squamous cell carcinoma which was incompletely excised (Fig. 1c). The patient received adjuvant topical mitomycin C and strontium plaque brachytherapy and remains recurrence free to date.

## Discussion

In our study from a single, UK-based tertiary pathology center, we received 2061 specimens submitted by ophthalmologists as possible pterygia over nearly 25 years. The rate of co-existent neoplasia in those specimens was only 0.6%, the vast majority of which were OSSN. This very low rate is consistent with some previous studies in geographic regions with lower levels of UVB exposure, despite our diverse population [5]. Studies which, like ours, included clinically suspicious lesions, found concomitant rates of OSSN in 0.3–9.8% of pterygium specimens, with higher rates in areas with higher UVB exposure [7–9].

In contrast, studies which excluded atypical lesions, found concomitant OSSN rates of 0–5% [5, 10]. Segev et al. found no OSSN in submitted pterygia samples, despite studying a population in a country of high UVB exposure, due in part to their exclusion of lesions which were considered suspicious clinically [6].

In our study, six cases had clinically atypical features, though only two cases were excised with full oncology precautions (wide margins, no touch technique and adjuvant cryotherapy). If we had excluded these pre-operatively suspicious lesions, the rate of incidental neoplasia would become only 0.3% in our study population. The proportion of the 2049 pterygium specimens without co-existent OSSN that had atypical features pre-operatively is unfortunately unknown.

In the three cases in the present study that had invasive neoplasia, one was found to be a melanoma. In pale skinned individuals with lightly pigmented irides, conjunctival melanoma can mimic OSSN [17]. It is important to bear this differential diagnosis in mind when the clinical features of a pterygium are atypical.

In two cases the “pterygium” turned out to be squamous cell carcinoma. The ethnicity of these were South Asian and African. Individual risk factors such as lifetime UV light exposure, immunosuppression or smoking history are not known in these cases. Both cases had clinically atypical features for a simple pterygium (nodule with inflammation or a gelatinous appearance). We would therefore advocate histopathological analysis for excised pterygia that display clinically unusual features.

Although histopathological analysis is the gold standard test, technological advances may assist in differentiating pterygia from OSSN. Use of in the clinic of vital dyes such as 0.05% Toluidine Blue vital staining for detecting cellular abnormality is of limited value, with high sensitivity but low specificity for OSSN [18]. More promising is anterior segment optical coherence tomography (AS-OCT) in detecting anatomical abnormality that is compatible with OSSN over pterygium [19] Rigorous testing of such devices may herald an era of a non-invasive tests to diagnose OSSN, followed by topical treatments—a notion that is being studied and may gain wider acceptance with results in various populations.

Although we had incomplete data on lesion location in three of the 12 cases, we found nasal location to be most common, consistent with previous reports [20]. Oellers et al. found inferior lesions to be more common in suspected OSSN [7].

Limitations of our study include the retrospective methodology. Moorfields Eye Hospital NHS Foundation Trust has multiple geographically distributed satellite sites within Greater London which send their histology specimens to the Department of Eye Pathology, each site with variable access to imaging, and varying levels of expertise in dealing with ocular surface malignancies. This has prevented us accessing long term follow up data for patients, and pre-operative information in some cases. Further prospective study is warranted to examine the rate of coexistent neoplasia with standardization of which lesions are considered typical or atypical.

Overall, we found the prevalence of co-existent neoplasia in pterygium specimens to be 0.6% in a UK-based population, but if we excluded clinically atypical lesions, this rate falls to 0.3%. This calls into question the time-honored doctrine of sending all excised pterygia for histopathological examination. This information will be useful in shaping clinicopathological pathways, of which specimens require histopathological diagnosis, particularly if imaging such as the AS-OCT leads us to “in vivo” biopsy. Rationing histology, however, would risk missing the rare occurrence of neoplasia in otherwise bland looking lesions, such as patient 2 in the present study, delaying diagnosis and potentially increasing the risk of locoregional spread.

It is the authors’ belief that in the UK population, experienced ophthalmologists can be reassured that the clinically typical pterygium is likely to be just that, especially if adjunctive investigations such as AS-OCT become more commonplace and prove reassuring. Histology remains crucial where there is clinical concern or recurrence of a previously excised lesion. This study may influence future guidance for the indications for submitting non-suspicious pterygia for histopathological examination.

## Summary

### What was known before

- Pterygia and ocular surface neoplasia share risk factors and have been found to coexist in a minority of patients.
- Studies worldwide have found rates of coexistence to vary between 0% and almost 10%.
- Higher rates are found in countries with higher UV light exposure.

### What this study adds

- No studies have examined for coexistent neoplasia in pterygia specimens in Europe or the UK.
- This study indicates a very low rate (0.6%).
- If clinically suspicious lesions are not included in this study, the rate of “unexpected” concurrent neoplasia drops to 0.3%.
- This may influence future guidance as to which specimens require histopathological study.

## Data Availability

Data are available on request only due to ethical reasons. Enquiries can be directed to the corresponding author.
